# A Rabbit Model Study to Determine the Efficacy of a Prototype Corneal Endothelium Protector during Cataract Surgery

**DOI:** 10.1155/2017/6906139

**Published:** 2017-03-29

**Authors:** Annabel C. Y. Chew, Anita Chan, Monisha E. Nongpiur, Gary Peh, Veluchamy A. Barathi, Nyein C. Lwin, Charles Ong, Shamira Perera

**Affiliations:** ^1^Singapore National Eye Centre, Singapore; ^2^Singapore Eye Research Institute, Singapore; ^3^Yong Loo Lin School of Medicine, National University of Singapore, Singapore

## Abstract

*Purpose*. We evaluated the efficacy and safety of a mechanical device, the P-chute, in corneal endothelium preservation during phacoemulsification in a rabbit model. *Methods*. Twenty-four rabbits were randomly assigned into 2 groups. One eye of each rabbit underwent phacoemulsification that simulated the removal of a dense nucleus, with or without the P-chute. Serial slit-lamp examinations, anterior segment optical coherence tomography (ASOCT) scans, and specular microscopy were performed. Three rabbits from each group were sacrificed on postoperative days (PODs) 1, 5, 7, and 14. Histological analysis of the corneas was performed. *Results*. There was a trend towards lesser endothelial cell loss for the P-chute group at POD1 (4.9% versus 12.5%, *p* = 0.53), POD5 (10.4% versus 12.2%, *p* = 0.77), and POD7 (10.5% versus 17.2%, *p* = 0.52). There was no significant difference in the corneal thickness (*p* = >0.05) between the 2 groups. The insertion of the device was challenging. The use of the P-chute only added an extra 15% to the surgical time. *Conclusions*. There was a trend towards better endothelium preservation with the P-chute even though the results were not statistically significant. We believe that the device could be useful in certain surgical situations. Further work is needed to improve the device insertion.

## 1. Introduction

Cataract surgery using phacoemulsification can cause a well-documented decrease in the endothelial cell count, which can lead to bullous keratopathy. The rate of endothelial cell loss has been reported to be between 7 and 52.6% depending on the complexity of the case [1–5], and the endothelial loss continues from the central cornea at a rate of 2.5% per year 10 years after the surgery [4]. Risk factors associated with higher endothelial cell loss are shorter axial length, longer phacoemulsification time [2], higher amount of ultrasound energy used [3], presence of preexisting cornea guttata [4], brunescent cataract, increased age of patient, capsular rupture or vitreous loss during surgery [5], and having diabetes [6].

The current techniques used to preserve the endothelium during cataract surgery include more effective ultrasound via a torsional delivery process [7, 8], dispersive ophthalmic viscoelastic devices (OVDs) [9–11], and the use of the “soft-shell” technique consisting of a dual layer of viscoelastics: a dispersive viscoelastic to coat the corneal endothelium and a cohesive viscoelastic to maintain the anterior chamber. There are no mechanical devices yet.

**Figure 1:**
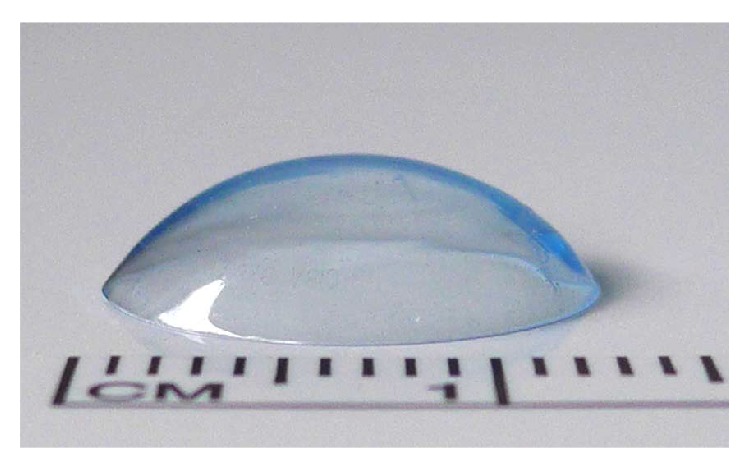
The design of the P-chute. This hydrogel contact lens would sit in the anterior chamber just beneath the cornea, surrounded by viscoelastic, and would serve as a physical barrier to protect the endothelium during phacoemulsification.

The P-chute (Figure 1), which is essentially a hydrogel contact lens, sits in the anterior chamber just beneath the cornea, surrounded by viscoelastic, and would serve as a more permanent physical protective barrier than routine viscoelastic alone. We hypothesized that the use of the P-chute will result in greater corneal endothelium preservation by reflecting fragments of nuclear material, dissipating ultrasound energy and heat away from the endothelium, and preventing the balanced salt solution (BSS) flow from removing the viscoelastic already protecting the endothelium. The primary objective of this study was to evaluate the efficacy and safety of the use of the P-chute on the corneal endothelial cells during phacoemulsification in a rabbit model. The primary outcome measure was percentage reduction in endothelial cell density postsurgery on specular microscopy. Secondarily, we assessed the ability to integrate the use of the P-chute into the phacoemulsification process.

## 2. Materials and Methods

### 2.1. Part I: Choosing the Hydrogel Contact Lens Material

The following experimental processes conformed to the tenets of the Declaration of Helsinki, and written consent was obtained from the next of kin of all deceased donors regarding the use of the corneas for research. This study was approved by the institutional review board of the Singapore Eye Research Institute and Singapore National Eye Centre.

Research-grade human cadaveric corneoscleral tissues were procured from the Lions Eye Institute for Transplant and Research Inc. (Tampa, FL, USA). Primary human corneal endothelial cells (HCECs) were isolated from these tissues and propagated to the second passage using a dual media approach as described [12]. At the third passage, HCECs were dissociated and seeded at a density of 2000 cells per mm^2^ on three different contact lenses to assess the cellular compatibility between the cultured HCECs and the various contact lenses. The contact lenses accessed include Air Optix (Alcon, USA), 1-day Acuvue (Johnson & Johnson Vision Care Inc., USA), and Acuvue Oasys (Johnson & Johnson Vision Care Inc., USA). Controls were HCECs seeded at the same cellular density onto glass coverslips.

We then performed endothelial cell staining with a calcein-AM/ethidium homodimer-1 (EthD-1) viability/cytotoxicity assay (Molecular Probes, Eugene, OR) to assess corneal endothelial cell viability. Briefly, the components of the viability/cytotoxicity assay were prepared, added to the corneal endothelial cells grown on the contact lens and glass coverslips, and incubated for 30 minutes at room temperature in the dark. The stained live samples were then viewed under an upright fluorescence microscope (Nikon Ti-Eclipse; Nikon Corp., Tokyo, Japan).

### 2.2. Part II: Evaluating the Efficacy of the P-Chute

#### 2.2.1. Surgical Procedures

The animal protocol of the study adhered to the Association for Research in Vision and Ophthalmology Statement for Use of Animals in Ophthalmic Vision and Research and was approved by the institutional review board and ethics committees of the Singapore National Eye Centre and Singapore Eye Research Institute.

Twenty-four female New Zealand white rabbits aged 5 to 6 months old and weighing between 3 and 3.5 kg were used for this study. The 24 rabbits were randomly assigned and equally divided into 2 study groups. Their eyes were examined under the slit lamp before the surgical procedures and were found to be unremarkable. Only the right eyes of the rabbits were used for the study. Group A had phacoemulsification surgery with the P-chute (*n* = 12), and group B had phacoemulsification surgery without the P-chute (*n* = 12).

Thirty minutes before the surgery, the pupil of each eye was dilated with topical minims phenylephrine hydrochloride 2.5% (Bausch and Lomb) and tropicamide 1% (Bausch and Lomb). The rabbits were anesthetized with an intramuscular injection of ketamine (40 mg/kg), xylazine (4 mg/kg), and topical xylocaine before the surgery. The periocular area was cleaned with povidone-iodine 10%. A wire lid speculum was placed to separate the eyelids, and topical povidone-iodine 5% was instilled onto the ocular surface for a few minutes prior to the surgery. An operating microscope was positioned over the eye undergoing surgery. The same surgeon operated on all the rabbits, using a standardized aseptic surgical technique simulating the extraction of a dense nucleus.

A 2.65 mm clear corneal incision was made in the superior quadrant using a keratome, and a 1.0 mm wide paracentesis was created 3 clock hours away to accommodate a chopper. Viscoelastic (Healon, AMO, Illinois, USA) was injected into the anterior chamber. Continuous curvilinear capsulorhexis and hydrodissection were performed. More viscoelastic was injected into the anterior chamber. The P-chute was rinsed with BSS, folded into one-third with the curvature of the contact lens orientated downwards, and pushed through the main corneal incision with Kelman-McPherson forceps. The P-chute was unfolded using a cannula. Phacoemulsification was performed using the Whitestar signature machine (AMO, Illinois, USA). As the rabbits had clear crystalline lenses, the lenses were removed using mainly aspiration (maximum ultrasound energy 10%, vacuum 300 mmHg, aspiration flow rate 30 cc/min, and bottle height 76 cm). The intraoperative phacoemulsification parameters were standardized in all eyes. To simulate the removal of a dense nucleus, the phacoemulsification probe was then placed in the anterior chamber and ultrasound energy 40% applied continuously for additional 5 minutes. Viscoelastic was injected into the anterior chamber prior to the removal of the P-chute, and the P-chute was removed by pulling it out through the main corneal incision with a pair of toothed forceps. The viscoelastic was then removed using aspiration (vacuum 500 mmHg, aspiration flow rate 30 cc/min). The corneal wounds were sutured with 10/0 nylon.

The surgery was repeated in the rabbits in group B, without the use of the P-chute.

#### 2.2.2. Clinical Evaluation

Detailed clinical examinations were performed by masked laboratory animal technicians preoperatively and postoperatively on day 1, day 5, day 7, and day 14 on anesthetized rabbits. The examinations included slit-lamp examination (FS-3V Zoom Photo Slit Lamp, Nikon, Japan) to check the degree of cornea clarity and intraocular inflammation, high-resolution anterior segment optical coherence tomography (ASOCT) scans of the cornea and anterior segment (Visante OCT, Carl Zeiss Meditec Inc., Dublin, California, USA), and in vivo endothelial cell count (ECC) using specular microscopy (Konan ROBO noncon specular microscope NS9900, Konan Medical Inc., Hyogo, Japan). Central corneal thickness (CCT) measurements were obtained using the measurement calipers provided by the Visante software, and the mean corneal thickness measurements in the central 6 mm zone were obtained from the pachymetry map. Postoperatively, all rabbits received topical tobradex 3 hourly (Alcon Laboratories, Fort Worth, Texas, USA) in the right eyes during the entire duration of follow-up.

**Figure 2:**
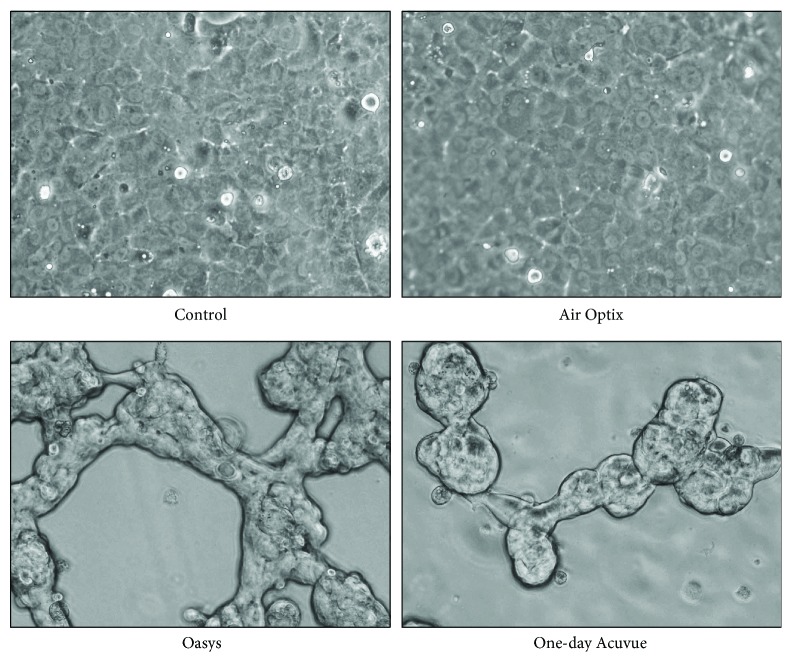
The results of seeding the cultivated HCECs on glass coverslips (as controls) and 3 different contact lenses. The HCECs were seen adhering and growing well as a monolayer on the Air Optix contact lens. The HCECs could not adhere well to the other surfaces of the contact lenses tested and instead formed sporadic cellular clusters.

Three rabbits from each group were sacrificed on postoperative day 1, day 5, day 7, and day 14 (Figure 2). The rabbits were sacrificed with an intravenous injection of sodium pentobarbital (100 mg/kg). The rabbit eyes were enucleated, and the corneas with a 2 mm rim of sclera were removed with a blade and scissors and fixed in 10% buffered formaldehyde (pH 7.0) for histology.

#### 2.2.3. Corneal Histopathology

The rabbit corneal specimens were bisected and embedded in paraffin, sectioned at 4 *μ*m, and stained with hematoxylin and eosin staining using standard techniques from the diagnostic histopathology laboratory of Singapore General Hospital. Cross-sectional analysis of the central full-thickness cornea (epithelium, stroma, Descemet's membrane, and endothelial cells) was performed using histological analysis. The stroma was reviewed for the presence of inflammatory cells (lymphocytes, neutrophils, and macrophages) and corneal edema. The number of endothelial cells was assessed using a 40× objective (field diameter of 0.55 mm, Olympus BX43 Light microscope) and was counted linearly on this field on 3 adjacent sections and the value averaged.

#### 2.2.4. Statistical Analysis

Statistical analysis was performed using the statistical package IBM SPSS Statistics for Windows (Version 22.0; IBM Corp., Armonk, NY, USA). Comparisons of the mean baseline values of CCT and ECC between the two groups were performed using the Mann-Whitney *U* test. Repeated measures linear mixed models analysis was conducted to compare the effect of P-chute during phacoemulsification on CCT, corneal thickness in the central 6 mm zone, and ECC in the 2 groups at the following time points: preoperatively and on postoperative days 1, 5, 7, and 14. As there was a significant difference in the preoperative ECC between the two groups, we assessed the percentage change in ECC at each postoperative time point compared to preoperative value using *t*-test and in the repeated measure analysis. The percentage change was calculated as ([{Preop ECC–postop ECC}/Preop ECC]^∗^100). The endothelial cell counts from the histology sections were also compared. The data was presented as mean ± SD (SEM). The significance was set at *p* < 0.05 for this study.

## 3. Results

### 3.1. Part I: Choosing the Hydrogel Contact Lens Material

**Figure 3:**
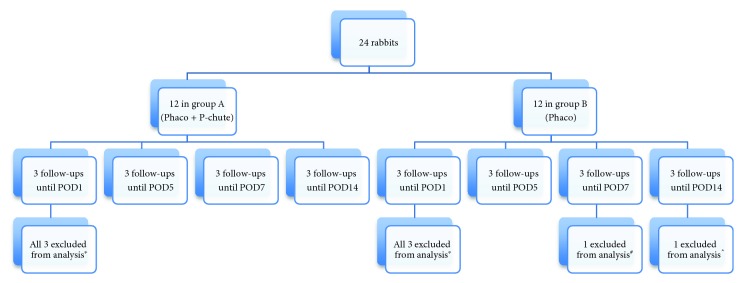
The flow chart of the clinical course of the rabbits. There were 12 rabbits in each group. ^∗^The first 6 rabbits were excluded from the result analysis due to the learning curve of the surgical procedure. ^#^Another rabbit whose pupil became small intraoperatively (followed up until POD7) and ^^^another rabbit in which the P-chute was inserted upside-down and scrapped against the corneal endothelium during the unfolding process (followed up until POD14) were also excluded from the result analysis.

Cultivated HCECs were able to adhere, grow well, and form an intact continuous monolayer when seeded onto Air Optix. Interestingly, when HCECs were seeded onto 1-day Acuvue or Acuvue Oasys, the cell could not adhere well to those surfaces to form a monolayer, but instead formed sporadic cellular clusters (Figure 3).

As HCECs were unable to adhere well on the 1-day Acuvue and Acuvue Oasys, the HCECs on these 2 contact lenses were excluded from the viability/cytotoxicity assay testing. The percentage of viable endothelial cells was over 99% in both the Air Optix and the glass coverslip groups.

Based on the results from our preliminary study, we chose to use the Air Optix for our study. The Air Optix is a silicone hydrogel contact lens, which is transparent, soft, inert, easy to mold, and does not disintegrate. The rabbit corneas have an average horizontal diameter of 15 mm and an average vertical diameter of 13.5 to 14 mm and an anterior chamber width of 13 mm [13, 14]. We used the contact lenses with a base curve of 8.6 mm, refractive power of 0 D, and center thickness of 0.08 mm and trephined them from 14.5 mm to a diameter of 10 mm to fit into the anterior chamber of rabbits' eyes.

### 3.2. Part II: Evaluating the Efficacy of the P-Chute

#### 3.2.1. Surgical Procedures

The P-chute did not affect the visualization or working space within the anterior chamber. The folding and insertion of the P-chutes were challenging as the contact lenses were very soft and the rabbits' anterior chambers were very shallow. Some of the P-chutes were not able to unfold fully in the anterior chamber until the crystalline lenses were debulked to create space in the anterior chamber. Two were inadvertently inserted upside-down, and 3 had significant movement in the anterior chamber during the surgery. In all cases, the removal of the P-chutes was straightforward. In one eye, the pupil became very small intraoperatively. The average surgical time in group A was 25 ± 1.15 (SEM 0.44) minutes and that in group B was 21.28 ± 1.21 (SEM 0.40) minutes. The surgeries were performed by a trainee surgeon with 5 years surgical experience on 400 phacoemulsification surgeries on human eyes (AC). Even so, the different dimensions of the rabbit eyes necessitated a steep learning curve and the first 3 eyes of each group were excluded.

#### 3.2.2. Clinical Evaluation

All eyes did not show any significant intraocular inflammation postoperatively. There was corneal edema in most of the eyes initially, which was resolved on postoperative day 14.

We excluded the first 6 rabbits from the result analysis due to the learning curve of the surgical procedure; all 6 rabbits were followed-up until postoperative day 1. We also excluded the rabbit whose pupil became small intraoperatively and another rabbit in which the P-chute was inserted upside-down and scrapped against the corneal endothelium during the unfolding process. The other eye in which the P-chute was inserted upside-down was included in the result analysis as there was not any significant corneal trauma during the unfolding process. Figure 2 shows the flow chart of the clinical course of the rabbits in group A and group B.

Table 1 shows the preoperative mean CCT, corneal thickness measurement in the central 6 mm zone, and ECC. The mean ECC was lower in group A compared to group B (2510 ± 297 cells/mm^2^ (SEM 112) versus 2980 ± 100 cells/mm^2^ (SEM 33), *p* = 0.001). Central corneal thickness (363.4 ± 19.4 *μ*m in group A (SEM 7.3) versus 367.3 ± 14.1 *μ*m in group B (SEM 4.7), *p* = 0.75) and the corneal thickness measurement in the central 6 mm zone (357.4 ± 10.0 *μ*m in group A (SEM 3.8) versus 364.4 ± 17.3 *μ*m in group B SEM 5.8), *p* = 0.17) were not significantly different between the 2 groups preoperatively.

Table 2 shows the postoperative percentage endothelial cell loss at various postoperative time points and ECC by histological analysis. There was a trend towards lower percentage of endothelial cell loss for group A at POD1 (4.9% in group A versus 12.5% in group B, *p* = 0.53), POD5 (10.4% in group A versus 12.2% in group B, *p* = 0.77), and POD7 (10.5% in group A versus 17.2% in group B, *p* = 0.52), even though the difference was not significant.

Controlling for repeated measures at 5 time points and using linear mixed models (Table 3), we found that there were no significant differences in the CCT (*p* = 0.07) and mean corneal thickness in the central 6 mm zone (*p* = 0.19) between the 2 groups (estimate, −64.4; 95% confidence interval (CI), −134.2 and 5.4 for CCT and estimate, −27.7; 95% CI, −69.9 and 14.5 for mean corneal thickness in the central 6 mm zone). There was also no significant difference for percentage change in ECC between the 2 groups (*p* = 0.56; estimate, 3.89; 95% CI, −10.52 and 18.31).

#### 3.2.3. Corneal Histopathology

**Figure 4:**
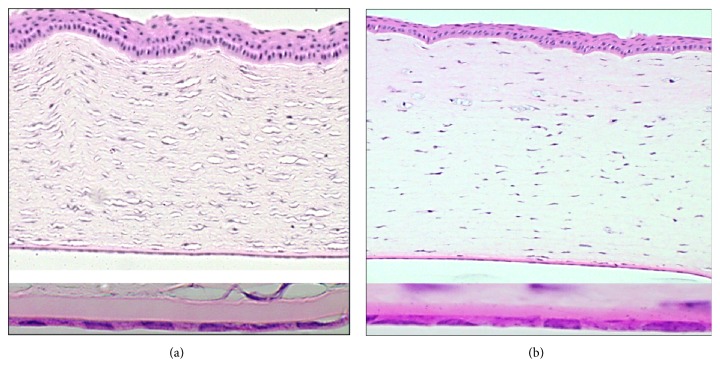
The histology of the cornea of a rabbit from group A and that of a rabbit from group B (hematoxylin and eosin, HE stains). (a) Image above, hematoxylin and eosin (HE), 20× magnification: the full-thickness cornea demonstrating the lack of corneal edema and inflammatory cells. Image below, HE, 40× magnification demonstrating the cross-sectional morphology of the endothelial cells and Descement's membrane. (b) Image above, hematoxylin and eosin (HE), 20× magnification: the full-thickness cornea demonstrating the lack of corneal edema and inflammatory cells. Image below, HE, 40× magnification demonstrating the cross-sectional morphology of the endothelial cells and Descement's membrane. No significant differences could be detected morphologically between the 2 groups, in particular the morphology of the endothelial cells.

The histology was reviewed by a histopathologist who was masked to the surgical procedures that were performed. The histological features of the 2 groups of corneas were similar, and the histopathologist could not discern any significant difference between the 2 groups (Figure 4). There was no significant corneal edema in both groups. Inflammatory cells (neutrophils, lymphocytes, and macrophages) were also not seen. The morphology of the endothelial cells did not show evidence of atrophy or apoptosis. The mean number of endothelial cells per field diameter of 0.55 mm was 31.6 ± 1.5 (SEM 0.6) in group A and 32.5 ± 1.5 (SEM 0.5) in group B (*p* = 0.24) (Table 2).

## 4. Discussion

There was a trend towards lesser endothelial cell loss in the group with the P-chute until postoperative day 7, with a 6.7% lesser endothelial cell loss, even though the results were not significant. This could be due to the small sample size, and that we were not able to obtain clear specular microscopy images in some eyes in the postoperative course due to the significant corneal edema. The results at postoperative day 14 were difficult to interpret due to the variable amount of rabbit endothelial regeneration [15]. To minimize any effects from endothelial regeneration, we sacrificed all the rabbits in the study at 2 weeks postoperation or earlier, since it would take at least 3 weeks to return to preinjury endothelial cell density and corneal thickness [16].

We encountered technical difficulties with the insertion of the P-chute into the extremely shallow rabbits' anterior chambers, which could have resulted in endothelial cell loss. This could be improved with a customized injector, or using a pull-through technique similar to that employed in Descemet's stripping endothelial keratoplasty (DSEK). The use of the P-chute added an extra 15% of surgical time to the operation.

When the P-chute is inserted the correct way up, there is little rotatory or other movements. This indicates that the specific 8.6 mm base curve of the P-chute is important in maintaining its position and allowing currents to move inside the eye without the displacement of the P-chute or the viscoelastic. Though to assess this scientifically, the viscoelastic anterior to the chute should be dyed or labeled in some way so as to quantify that the device was in fact useful in retaining the viscoelastic immediately adjacent to the endothelium.

Our current surgical model did not simulate the phacoemulsification of a dense nucleus well. Rabbits' lenses were soft, and there was an absence of multiple hard nuclear fragments that could mechanically scratch the endothelium such as those present in human phacoemulsification surgery. However, the model adequately simulated human phacoemulsification surgery of a soft cataract with an extremely shallow anterior chamber with a moderately experienced surgeon. Further work in a more accurate model mimicking human phacoemulsification surgery with a denser cataract should be done; the next step would be to gradually increase ultrasound energy levels until a difference between the two groups can be noticed.

Torsional ultrasound has been shown to reduce the amount of endothelial cell loss by around 3% compared to conventional ultrasound [8]. A meta-analysis evaluating the protective effect of OVDs on endothelial cell density up until 2007 concluded that viscoadaptive OVDs were the best option, followed by the “soft-shell” technique. However, the absolute differences in the endothelial cell loss between the various OVDs at 3 months after surgery were small (<100 cells/mm^2^) [9]. The newer viscodispersive OVDs may offer improved endothelium protection, with lesser endothelial cell loss reported at 3 months postphacoemulsification compared to viscoadaptive OVDs (1.8% ± 8.7% versus 3.8% ± 8.3%, *p* = 0.0358).

We believe that with an improved insertion technique, the P-chute may be useful in cases where one needs to perform phacoemulsification more anteriorly due to a floppy posterior capsule, or in the presence of a small pupil or an anterior capsular tear.

The limitations in our study included the following: the small sample size, the technical difficulties with the P-chute insertion, the current rabbit model not being a good surgical surrogate for the phacoemulsification of actual dense nuclei, and a regenerating endothelial model.

In summary, we evaluated the first use of a mechanical device to protect the corneal endothelium during phacoemulsification in a rabbit model. There was a trend towards better endothelial preservation with the use of the P-chute even though the results were not statistically significant. We believe that the device could be useful in certain surgical situations. Further work is needed to improve the insertion of the device.

## Conflicts of Interest

The authors declare that there is no conflict of interest regarding the publication of this paper.

## Figures and Tables

**Table 1:** Preoperative mean CCT, corneal thickness in the central 6 mm zone, and ECC.

	Group A (*n* = 9)	Group B (*n* = 7)	*p* value^∗^
CCT (*μ*m)	363.4 ± 19.4 (SEM 7.3)	367.3 ± 14.1 (SEM 4.7)	0.75
Mean corneal thickness in the central 6 mm zone (*μ*m)	357.4 ± 10.0 (SEM 3.8)	364.4 ± 17.3 (SEM 5.8)	0.17
ECC (cells/mm^2^)	2510 ± 297 (SEM 112)	2980 ± 100 (SEM 33)	0.001

^∗^Mann-Whitney *U* test.

**Table 2:** Postoperative percentage endothelial cell loss and mean number of endothelial cells by histological analysis.

	Mean preoperative ECC (cells/mm^2^)	Percentage endothelial cell loss (%)				Mean ECC by histological analysis (cells/field diameter)
		POD1	POD5	POD7	POD14	
Group A (*n* = 9)	2510 ± 297 (SEM 112)	4.9 ± 9.2% (SEM 6.50)	10.4 ± 12.4% (SEM 5.53)	10.5 ± 9.7% (SEM 4.87)	9.8 ± 0.52% (SEM 0.37)	31.6 ± 1.5 (SEM 0.6)
Group B (*n* = 7)	2980 ± 100 (SEM 33)	12.5% ± 14.9% (SEM 6.06)	12.2% ± 10.0% (SEM 3.55)	17.2% ± 18.0% (SEM 7.35)	4.7% ± 11.4% (SEM 6.60)	32.5 ± 1.5 (SEM 0.5)
*p* value	0.001	0.53	0.77	0.52	0.59	0.24

**Table 3:** Repeated measures linear mixed models for comparison of the effect of P-chute in the two groups.

	CCT				Corneal thickness in the central 6 mm zone				ECC (percentage change from preop)			
Parameter	Estimate	Sig.	95% CI		Estimate	Sig.	95% CI		Estimate	Sig.	95% CI	
Intercept	402.86	0.000	282.96	522.75	394.90	0.000	333.79	456.00	10.23	0.12	−3.02	23.47
Preop	−0.99	0.99	−130.14	128.14	−17.94	0.58	−82.14	46.27				
Postop day 1	234.75	0.001	105.61	363.89	96.79	0.005	29.89	163.69	1.03	0.86	−10.91	12.98
Postop day 5	46.88	0.47	−83.13	176.88	78.79	0.02	15.40	142.18	0.39	0.94	−10.08	10.87
Postop day 7	46.37	0.49	−89.34	182.08	58.83	0.06	−2.92	120.57	1.52	0.73	−7.40	10.44
Postop day 14	Ref	—	—	—	Ref	—	—	—	Ref	—	—	—
Group B	−64.41	0.07	−134.23	5.40	−27.71	0.19	−69.90	14.48	3.89	0.56	−10.52	18.31
Group A	Ref	—	—	—	Ref	—	—	—	Ref	—	—	—

The table shows the comparison of the CCT, corneal thickness measurement in the central 6 mm zone, and ECC preoperatively and on postoperative days 1, 5, 7, and 14 using repeated measures linear mixed models.
